# Mangiferin Reduces the Inhibition of Chondrogenic Differentiation by IL-1β in Mesenchymal Stem Cells from Subchondral Bone and Targets Multiple Aspects of the Smad and SOX9 Pathways

**DOI:** 10.3390/ijms150916025

**Published:** 2014-09-11

**Authors:** Jeong-Eun Huh, Pil-Seong Koh, Byung-Kwan Seo, Yeon-Chul Park, Yong-Hyun Baek, Jae-Dong Lee, Dong-Suk Park

**Affiliations:** 1 East-West Bone & Joint Research Institute, Kyung Hee University, 149, Sangil-dong, Gangdong-gu, Seoul 134-727, Korea; E-Mail: jehuh2551@hanmail.net; 2 Department of Acupuncture and Moxibustion, College of Oriental Medicine, Kyung Hee University, 1, Hoegi-dong, Dongdaemun-gu, Seoul 130-701, Korea; E-Mails: webs0905@naver.com (P.-S.K.); ljdacu@khumc.or.kr (J.-D.L.); 3 Department of Acupuncture and Moxibustion, Kyung Hee University Hospital at Kangdong, 149, Sangil-dong, Gangdong-gu, Seoul 134-727, Korea; E-Mails: sbkacu@khu.ac.kr (B.-K.S.); icarus08@hanmail.net (Y.-C.P.); byhacu@khu.ac.kr (Y.-H.B.)

**Keywords:** mangiferin, chondrogenic differentiation, mesenchymal stem cells, Smads, sex-determining region Y–box (SRY-box) containing gene 9 (SOX9)

## Abstract

Mangiferin is a natural immunomodulator found in plants including mango trees. The effects of mangiferin on chondrogenesis and cartilage repair have not yet been reported. This study was designed to determine the effect of mangiferin on chondrogenic differentiation in IL-1β-stimulated mesenchymal stem cells (MSCs) from subchondral bone and to explore the mechanisms underlying these effects. MSCs were isolated from the subchondral bone of rabbit and treated with mangiferin alone and/or interleukin-1β (IL-1β). Mangiferin induced chondrogenic differentiation in MSCs by upregulating transforming growth factor (TGF)-β, bone morphogenetic protein (BMP)-2, and BMP-4 and several key markers of chondrogenesis, including sex-determining region Y–box (SRY-box) containing gene 9 (SOX9), type 2α1 collagen (Col2α1), cartilage link protein, and aggrecan. In IL-1β-stimulated MSCs, mangiferin significantly reversed the production of TGF-β, BMP-2, BMP-4, SOX9, Col2α1, cartilage link protein, and aggrecan, as well as matrix metalloproteinase (MMP)-1, MMP-13, and a disintegrin and metalloproteinase with thrombospondin motifs (ADAMTS5). Mangiferin upregulated the phosphorylation of Smad 2, Smad 3, Smad 1/5/8, and SOX9 in IL-1β-stimulated MSCs. In the presence of mangiferin, SOX9 siRNA suppressed the activation of Smad 2, Smad 3, Smad 1/5/8, aggrecan, and Col2α1 expression. In conclusion, mangiferin exhibits both chondrogenic and chondroprotective effects on damaged MSCs and mediates these effects by targeting multiple aspects of the Smad and SOX9 signaling pathways.

## 1. Introduction

Osteoarthritis (OA) is the most common musculoskeletal disease, and it imposes heavy social, medical, and financial burdens. Current strategies for OA treatment consist of suspending surgery, reducing pain and stiffness, and improving joint function. The commonly prescribed OA medications are non-steroidal analgesics, locally administered corticosteroids, and non-steroidal anti-inflammatory drugs (NSAIDs), which offer only symptomatic relief and eventually lead to surgical intervention [1]. Several novel therapeutic methods for the treating OA are being researched, and it is anticipated that these new therapies will soon enable us to halt the progression of this degenerative disease and restore cartilage homeostasis [2]. Subchondral bone is a target of current OA treatments, because subchondral bone changes in OA induce cartilage loss, and cartilage damage may, in turn, have a negative influence on subchondral bone [3,4]. A previous study showed that bone sclerosis caused by subchondral bone damage may precede cartilage degradation [5]. Furthermore, microfracture can damage the tissue down to the subchondral bone and induce bleeding, allowing mesenchymal stem cells (MSCs) derived from the subchondral bone to enter the defect. Therefore, subchondral bone tissue may be a promising therapeutic target in OA.

MSCs of subchondral bone are capable of differentiating into several different lineages and have been considered as a candidate cell source for tissue engineering and regenerative medicine [6,7]. Human subchondral cortico-spongious progenitor cells have cell surface antigens known to be typical of mesenchymal stem and progenitor cells, such as CD73, CD90, CD105, and CD166 [8]. Previously, we have characterized chondrogenic lineage development and have shown that the induction of typical chondrogenic marker genes from rabbit subchondral bone results in the deposition of cartilage matrix molecules, such as type II collagen and proteoglycan [9]. Despite this, cartilage regeneration is inefficient, and the fibrocartilage resulting from this process is structurally and functionally inadequate [5,8]. A possible explanation for this is that the ongoing inflammatory processes that occur during the course of OA result in higher levels of pro-inflammatory cytokines, such as IL-1β, which may impede the chondrogenic differentiation of cartilage resident progenitors [8,10]. Therefore, blocking the cartilage generation induced by pro-inflammatory cytokines could create a more suitable microenvironment for chondrogenesis of MSCs-like progenitors.

Chondrogenesis is a complex process, in which undifferentiated progenitor cells and numerous growth factors work in concert to induce chondrogenic differentiation [7,8,11]. Several cytokines and transcription factors, including transforming growth factor (TGF)-β, bone morphogenetic proteins (BMPs), fibroblast growth factor (FGF), parathyroid hormone-related peptide (PTHrP), SOX proteins, and Cbfa1, contribute to the chondrogenic process and/or to the control of bone morphogenesis [12,13]. TGF-β and BMPs have been involved in chondrogenesis mediated by Smad transcription factors, which bind to TGF-β receptors and are phosphorylated following binding to type II receptors [14,15]. Both BMP receptor-associated Smads (1, 5, and 8) and the TGF-β receptor-associated Smads (2 and 3) are released into the cytoplasm upon phosphorylation and form a complex with Smad4, which then translocates into the nucleus, where it regulates the expression of genes such as SOX9 [16,17]. SOX9 regulates the expression of a major cartilage matrix protein, type II collagen α1 (Col2α1), and enhances aggrecan gene activity in chondrocytes. These observations indicate that SOX9 plays a key role in chondrogenesis [14,15,16,17].

Mangiferin is a natural polyphenol compound commonly found in both mango and papaya, and it possesses many beneficial biological activities, including anti-oxidant, anti-tumor, anti-viral, anti-diabetic, and immunomodulatory activities [18,19,20,21]. Mangiferin was also reported to have anti-osteoclastogenic activity in the treatment and prevention of bone diseases [22]. Previous studies suggest that mangiferin may curb the over-activation of NF-κB and, therefore, may have potential as an alternative medicine for the treatment of tumors, inflammation, and osteolytic bone disease [18,19,20,21,22]. However, the effects of mangiferin on chondrogenic differentiation and cartilage repair have not yet been reported.

The aims of the present investigation were to evaluate whether mangiferin stimulates chondrogenic differentiation of MSCs from subchondral bone; to examine the effect of mangiferin on adverse catabolic and anabolic responses in IL-1β-stimulated MSCs; and to determine the mechanism of action of mangiferin on the targets of Smad and SOX9 signaling pathways.

## 2. Results and Discussion

### 2.1. Mesenchymal Stem Cells from Subchondral Bone Differentiate into Chondrogenic, Adipogenic, and Osteogenic Cells

**Figure 1:**
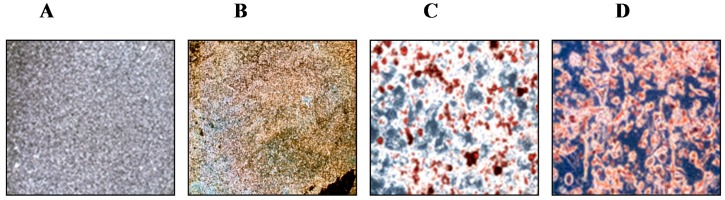
Histological analysis of mesenchymal stem cells from rabbit subchondral bone undergoing chondrogenic, adipogenic, and osteogenic differentiation. (**A**) control; (**B**) chondrogenic differentiation visualized by staining with alcian blue; (**C**) adipogenic differentiation visualized by staining with Oil Red O; and (**D**) osteogenic differentiation visualized by staining with Alizarin Red.

To evaluate the chondrogenic differentiation potential of MSCs from rabbit subchondral bone, cells were cultured for up to 14 days in pellet cultures under standard chondrogenic conditions. Histological analysis using the alcian blue stain showed that progenitor cells developed into a dense tissue, rich in viable cells and proteoglycan (Figure 1B). Adipogenic differentiated cells were visualized through the staining of lipid vacuoles by Oil Red O, and osteogenic cells were detected via the staining of mineralized matrix components with Alizarin red (Figure 1C,D). This study indicates that MSCs from the subchondral bone have a prominent chondrogenic, adipogenic, and osteogenic differentiation potential. In our previous study, MSCs from rabbit subchondral bone showed typical surface antigens, approximately 91%–98% of which were for CD105, CD73, and CD90. In addition to the pluripotency factors OCT4 and NANOG similary express, as do the adult bone marrow-derived MSCs that we examined. Cells were negative for the hematopoietic antigens CD3 and CD34, as well as for the common leukocyte antigen CD45 and the macrophage antigen CD11b [9,10]. In line with the previous studies, these data suggest that MSCs of subchondral bone are similar to MSCs from many other tissues and have potential as a substitute route for cartilage repair.

### 2.2. Mangiferin Enhances Chondrogenic Differentiation and Influences Induction of Cartilage Phenotypic Markers and Growth Factors

**Figure 2:**
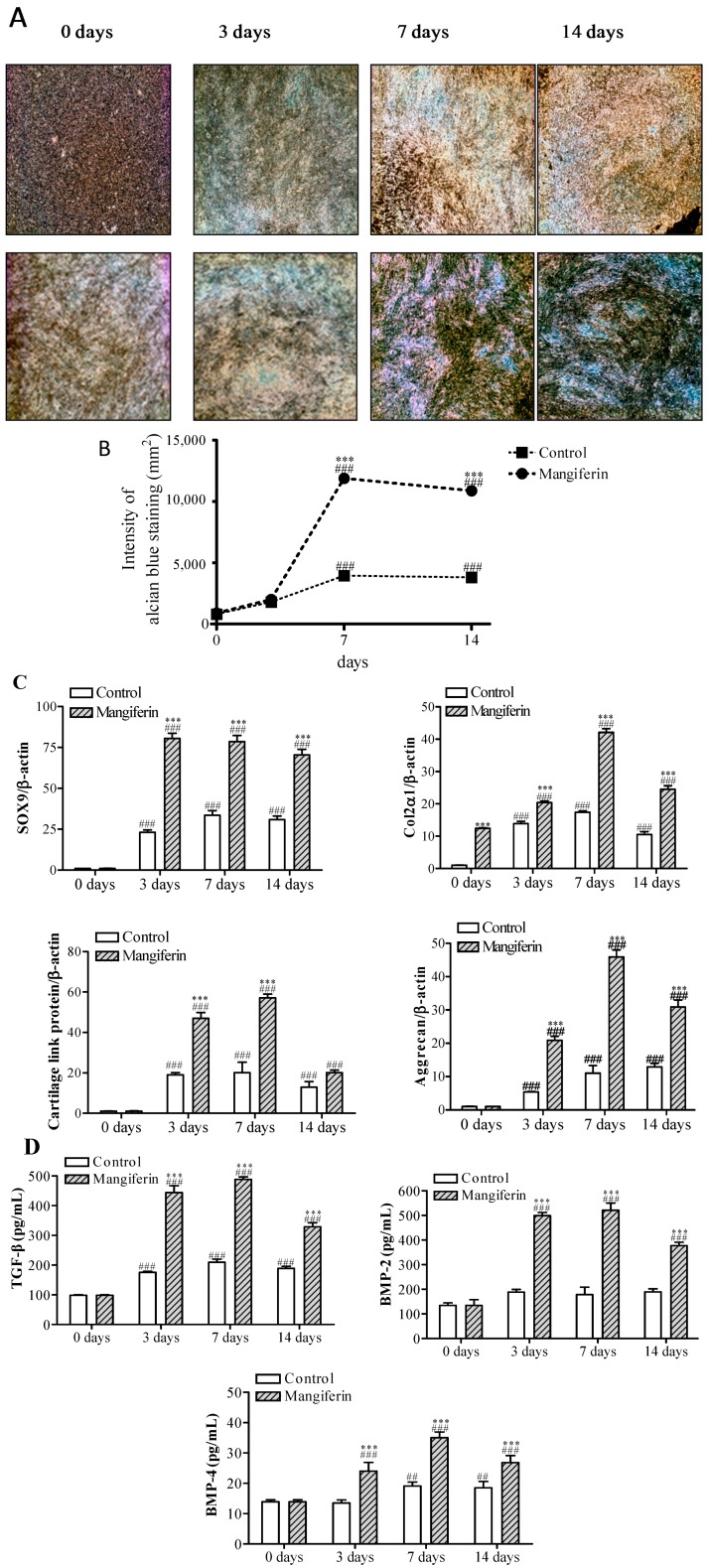
Effects of mangiferin on chondrogenic differentiation in mesenchymal stem cells (MSCs). (**A**) Histological analysis by alcian blue staining during chondrogenesis of MSCs pellet culture. Chondrogenic differentiation of MSCs was induced by mangiferin at 10 µM. Cells were then stained with alcian blue. Cartilage nodules were observed following alcian blue staining at 3, 7, and 14 days after culturing under chondrogenic conditions (**upper panel**) or in the presence of 10 µM mangiferin (**lower panel**); (**B**) Line graph showing the staining intensity of alcian blue; (**C**) The mRNA expression of chondrogenic markers in MSCs. The level of sex-determining region Y–box (SRY-box) containing gene 9 (SOX9), type 2α1 collagen (Col2α1), cartilage link protein, and aggrecan were measured by real time RT-PCR at days 3, 7, and 14, and normalized relative to β-actin; and (**D**) The level of transforming growth factor (TGF)-β, bone morphogenetic protein (BMP)-2, and BMP-4 measured using enzyme-linked immunosorbent assay (ELISA) during chondrogenesis of MSCs. Results are from at least three separate experiments, and each bar represents the mean ± standard error of mean (SEM). ^##^ *p* < 0.01 and ^###^ *p* < 0.001 compared with 0 day. *** *p* < 0.001 compared with control.

To evaluate the effect of mangiferin on chondrogenesis, subchondral bone-derived MSCs were placed in pellet culture and treated with 10 µM mangiferin. After 7 days in culture, treatment with mangiferin resulted in the stimulation of chondrogenic differentiation and a 3.9-fold increase in alcian blue nodule intensity at 14 days, compared with the control cells (Figure 2A,B). To assess chondrogenic differentiation in MSCs cultures on the molecular level, the mRNA expression levels of SOX9, Col2α1, cartilage link protein, and aggrecan were determined by performing real time RT-PCR, and the levels of TGF-β, BMP-2, and BMP-4 were measured via the enzyme-linked immunosorbent assay (ELISA) assay. Mangiferin significantly induced the expression of phenotypic markers of chondrogenesis (SOX9, Col2α1, cartilage link protein, aggrecan), with a concordant upregulation of growth factors (TGF-β, BMP-2, BMP-4) (Figure 2C). Compared with control cells, the level of SOX9 increased by 3.3-, 2.4-, and 2.3-fold at days 3, 7 and 14, respectively. During the culture period, SOX9 was continuously expressed until day 14, peaking at day 3 (Figure 2C). At day 7, the greatest increases in the levels of Col2α1 (2.5-fold), cartilage link protein (3.1-fold), and aggrecan (3.7-fold) were observed following mangiferin treatment; all levels decreased slightly on day 14 (Figure 2C). Mangiferin markedly increased the levels of TGF-β (2.5- to 2.3-fold), BMP-2 (2.7- to 2.9-fold), and BMP-4 (1.8- to 1.8-fold) at days 3 and 7, respectively, compared with control; levels were reduced slightly at day 14 (Figure 2D). These results indicate that mangiferin induces chondrogenic differentiation by increasing SOX9 expression over several days and influences chondrogenic regulation induced by TGF-β, BMP-2, and BMP-4. SOX9 is responsible for the expression of some of the key genes in chondrogenesis [23,24,25,26]. Moreover, SOX9 regulates the expression of Col2α1, cartilage link protein collagen IXα1 (Col9α1), and aggrecan, which is the most abundant proteoglycan in cartilage [25,26]. Akiyama *et al.* [25] have shown that all osteo-chondroprogenitors stem from SOX9-expressing cells, confirming a principal role for SOX9 in chondrogenesis [26]. SOX9 is activated by the expression of TGF-β and BMP during very early events in chondrogenesis and, directly or indirectly, maintains their regulation during the differentiation and maturation of chondrocytes [27,28]. There is considerable crosstalk between the TGF and BMP signaling pathways, as evidenced by the synergistic effect of TGF-β and BMP-2 on Col2α1 and aggrecan mRNA expression [29,30]. The present study showed that mangiferin notably induced SOX9, Col2α1, cartilage link protein, aggrecan, and growth factors such as TGF-β, BMP-2, and BMP-4. Our results suggest that mangiferin enhances chondrogenesis by stimulating SOX9 and TGF-β/BMPs expression in MSCs from subchondral bone.

### 2.3. Mangiferin Reverses the Inhibition of IL-1β-Induced Chondrogenic Differentiation by Regulation of Anabolic and Catabolic Genes

**Figure 3:**
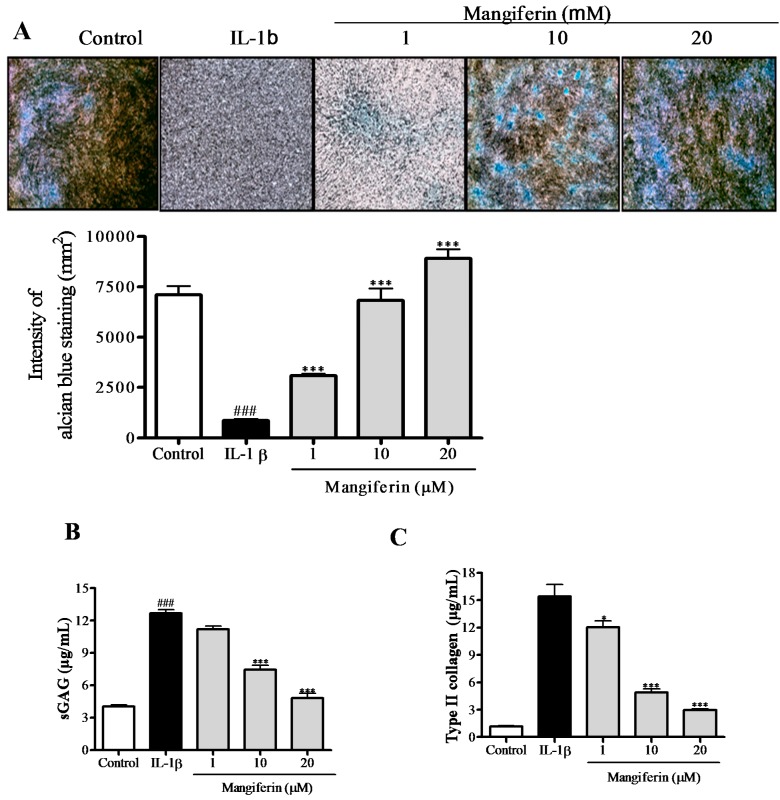
Effect of mangiferin on the chondrogenic differentiation of IL-1β-stimulated mesenchymal stem cells (MSCs). (**A**) Histological analysis of mangiferin by alcian blue staining shows recovery of chondrogenic differentiation in IL-1β-stmulated MSCs treated with mangiferin. The bar graph shows the intensity of alcian blue staining; (**B**,**C**) Inhibitory effect of mangiferin on sulfated glycosaminoglycan (sGAG) and type II collagen degradation in IL-1β-stimulated MSCs. The release of sGAG (**B**) and type II collagen (**C**) were shown as the cumulative release into the culture medium, collected at day 7 and measured by colorimetric analysis. Results were from at least three separate experiments, and each bar represents the mean ± SEM. ^###^ *p* < 0.001 compared with control. * *p* < 0.05 and *** *p* < 0.001 compared with IL-1β.

To examine the effects of mangiferin on IL-1β-induced MSCs-derived chondrocytes, we first evaluated proteoglycan contents and the release of glycosaminoglycan (GAG) and type II collagen by these cells. As shown in Figure 3A, IL-1β caused less area to be stained by alcian blue compared with control, while mangiferin treatment reversed the inhibitory effect of IL-1β on proteoglycan stained area. Figure 3A shows that mangiferin treatment dose-dependently increased the intensity of alcian blue staining compared with cells treated with IL-1β alone (Figure 3A). Comparison of the degradation of GAG and type II collagen between the mangiferin and IL-1β-treated groups showed that mangiferin at 10 and 20 µM significantly inhibited the release of GAG (1.7- and 2.6-fold, respectively) (Figure 3B) and reduced the degradation of type II collagen (3.1- and 5.3-fold, respectively) (Figure 3C).

**Figure 4:**
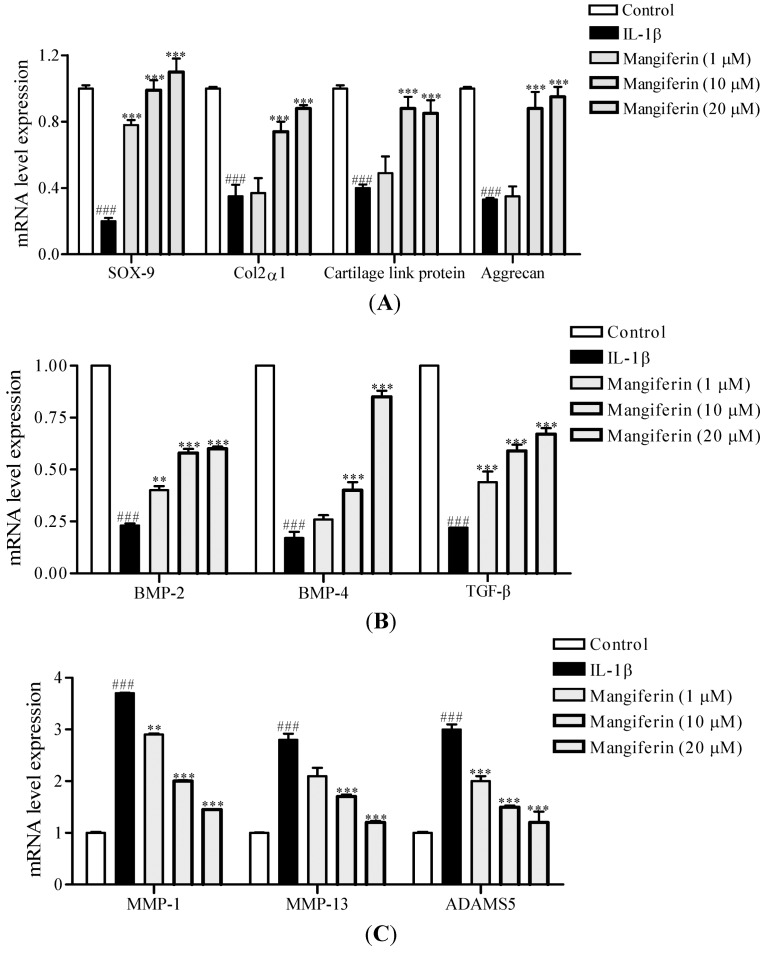
Effects of mangiferin on the expression of anabolic and catabolic genes in IL-1β-stimulated mesenchymal stem cells (MSCs). (**A**) mRNA expression levels of the chondrogenic markers SOX9, Col2α1, cartilage link protein, and aggrecan. MSCs pellet culture was treated with mangiferin (1, 10, and 20 µM) for 7 days. mRNA expression was measured by real time RT-PCR and normalized relative to β-actin; (**B**) mRNA expression levels of TGF-β, BMP-2, and BMP-4; and (**C**) mRNA expression levels of MMP-1, MMP-13, and ADAMS5. Data were obtained from three independent experiments and are represented as mean ± SEM. ^###^ *p* < 0.001 compared with control. ** *p* < 0.01, and *** *p* < 0.001 compared with IL-1β.

Next, we determined the effect of mangiferin on the level of anabolic (Col2α1, SOX-9, cartilage link protein, aggrecan, BMP-2, BMP-4, TGF-β) and catabolic (MMP-1, MMP-13, ADAMS5) genes, respectively. Mangiferin at 1, 10, and 20 µM increased the expression of SOX9 (3.9- to 5.5-fold), Col2α1 (1.0- to 2.5-fold), cartilage link protein (1.2- to 2.1-fold), and aggrecan (1.0- to 2.9-fold) compared with IL-1β-stimulated MSCs not treated with mangiferin (Figure 4A). Mangiferin treatment reversed the inhibition of expression of BMP-2 (1.6- to 3.5-fold), BMP-4 (1.6- to 3.8-fold), and TGF-β (2.0- to 3.1-fold) in a dose-dependent manner (Figure 4B). Mangiferin significantly inhibited the IL-1β-induced upregulation of MMP-1 (1.3- to 2.5-fold), MMP-13 (1.3- to 2.3-fold), and AMAMS5 (1.5- to 2.5-fold) expression (Figure 4C).

In general, IL-1β can stimulate the expression of not only proinflammatory mediators, but also stimulatory catabolic proteinases such as MMPs and ADAMTS [31,32]. IL-1β has been shown to significantly reduce basal cell migration and abrogate the stimulatory effect of growth factors in chondrogenic progenitor cells from cartilage tissue, which might lead to reduced cartilage regenerative potential [33]. Earlier studies reported that TGF-β1 stimulates chondrocyte synthetic activity and chondrogenesis of bone marrow-derived MSCs and decreases the catabolic activity of IL-1β [34]. Promising studies in rabbits have shown that TGF-β1 can enhance the repair of cartilage defects [35]. BMP-2 stimulates matrix synthesis and is capable of reversing chondrocyte dedifferentiation to some extent, as indicated by an increase in the synthesis of cartilage-specific type II collagen in dedifferentiated OA chondrocytes [12,36]. In a mouse model of IL-1β-induced cartilage degeneration, BMP-2 enhanced cartilage matrix turnover, as evidenced by increased aggrecan and type II collagen expression [37]. Thus, we suggest that mangiferin reduces IL-1β-induced inhibition of chondrogenic differentiation by regulation of anabolic factors and catabolic genes, indicating that mangiferin may stimulate cartilage regeneration in IL-1β-stimulated MSCs from subchondral bone.

### 2.4. Mangiferin Upregulates the Phosphorylation of Smad and SOX9 Signaling Pathways in IL-1β-Stimulated Mesenchymal Stem Cells (MSCs)

**Figure 5:**
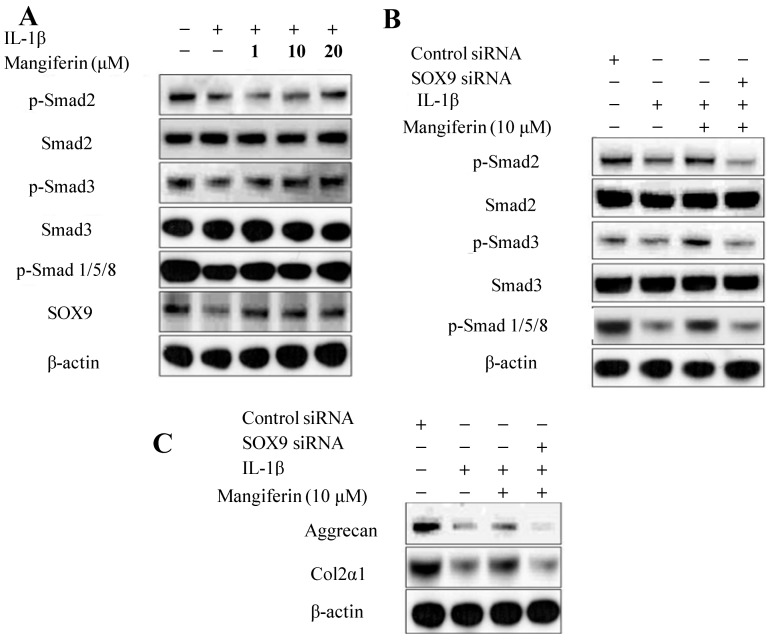
Effects of mangiferin on the phosphorylation of the Smad and SOX9 signaling pathways in IL-1β-stimulated mesenchymal stem cells (MSCs). (**A**) Mangiferin upregulates the phosphorylation of Smad 2, Smad 3, Smad 1/5/8, and the SOX9 signaling pathways. MSCs were prestimulated with IL-1β (5 ng/mL) for 1 h and then treated with the indicated dose of mangiferin for 72 h. Whole cell lysates were subjected to Western blotting with the indicated antibodies; (**B**,**C**) SOX9 deficiency inhibits the phosphorylation of Smad 2, Smad 3, Smad 1/5/8, aggrecan, and Col2α1. β-actin served as an internal control. MSCs were transfected with control siRNA or SOX9 siRNA and then treated with or without mangiferin for 72 h. Whole cell lysates were subjected to Western blotting with the indicated antibodies. siRNA targeting SOX9 was transfected into two different experiments.

We hypothesized that the effect of mangiferin on TGF-β and BMP expression and induction of chondrogenesis is likely to be related to the regulation of Smad signaling. To test this, we determined the involvement of mangiferin in the Smad and SOX9 signaling pathways. Western blot analysis of IL-1β-stimulated MSCs revealed that the chondrogenic differentiation process was disturbed due to the downregulation of phosphorylation of Smad 2, Smad 3, Smad 1/5/8, and SOX9. Mangiferin treatment increased the phosphorylation of Smad 2, Smad 3, and Smad 1/5/8 in IL-1β-stimulated MSCs (Figure 5A). Smads are associated with the TGF-β and BMP signaling pathways, which, in turn, are involved with MSCs differentiation [38,39,40,41]. Smad 2 and Smad 3 are known to mediate transcription processes in the TGF-β signaling pathway, and Smad 1/5/8 is known to mediate transcription in the BMP signaling pathway, leading to regulation of the transcription factor SOX9 [38,39,40]. SOX9 plays a key role in chondrogenesis and controls the expression of aggrecan and type II collagen [42,43]. To determine the involvement of SOX9 in the expression of aggrecan and Col2α1, IL-1β-stimulated MSCs were transfected with control siRNA or SOX9 siRNA and then treated with mangiferin. In the presence of mangiferin and SOX9 siRNA, phosphorylation of Smad 2, Smad 3, Smad 1/5/8, aggrecan, and Col2α1 was suppressed in IL-1β-stimulated MSCs (Figure 5B,C). This suggests that SOX9 is an important pathway for the chondrogenic differentiation process, and that mangiferin may act on SOX9 to normalize chondrogenic differentiation and chondrogenesis. Thus, the present results indicate that mangiferin affects chondrogenesis by stimulating the SOX9 and by inducing the phosphorylation of Smad 1/5/8 and Smad 2/3 signaling pathways.

## 3. Materials and Methods

### 3.1. Chemicals and Reagents

Mangiferin (purity: 97%, reference standard), alcian blue, Alizarin red S staining kit, Oil red O staining kit, ascorbic acid, glycerophosphate, dexamethasone, 3-isobutyl-1-methylxanthine, dimethyl sulfoxide, and insulin were purchased from Sigma-Aldrich (St Louis, MO, USA). Fetal bovine serum (FBS), fetal calf serum (FCS), antibiotics, Dulbecco’s modified Eagle’s medium (DMEM), TRIzol R reagent, and SDS-polyacrylamide gels were purchased from Gibco-BRL (now part of Invitrogen Corporation; Carlsbad, CA, USA). The real-time SYBR Green RT-PCR system kit was purchased from Bio-Rad (Roche Diagnostics, Mannheim, Germany). Hybond-C nitrocellulose membrane was purchased from Amersham Biosciences (Piscataway, NJ, USA).

### 3.2. Animals

Male New Zealand white rabbits (2.8–3.0 kg, 9–10 weeks), acquired from the animal experimental center at Kyung Hee University Medical Center (Seoul, Korea), were kept separately with water and food freely available. The room was light/dark (08:00–20:00 h light, 20:00–08:00 h dark) controlled and maintained at 21–24 °C. All experiments were carried out according to the Guiding Principles for the Care and Use of Laboratory Animals, and all procedures were approved by the Animal Care and Use Committee of Kyung Hee University Medical Center.

### 3.3. Cell Culture

Subchondral bone was obtained from the knee joint of New Zealand white rabbits. To gather MSCs, subchondral bone was cut into small fragments, washed with phosphate buffered saline, and partially digested with 256 U/mL type I collagenase (Sigma-Aldrich) for 4 h at 37 °C. The supernatant was discarded and the remaining fragments were placed in culture flasks. The fragments were then cultured in DMEM (Gibco-BRL) containing 10% heat-inactivated FBS and an antibiotic mixture (100 units/mL penicillin base and 100 μg/mL streptomycin) at 37 °C in a humidified atmosphere. Cells that reached 80%–90% confluence were passaged using trypsin-EDTA in PBS (0.05% *v*/*v*, Gibco-BRL) and expanded into plates in passage 1. The medium was replaced every 3 days. Pellets of MSCs were treated with mangiferin (1, 10, and 20 µM) in the absence or in the presence of IL-1β (5 ng/mL) for 3, 7, and 14 days of culture.

### 3.4. Assessment of Mesenchymal Stem Cell Differentiation Potential from Subchondral Bone

To induce chondrogenic differentiation, aliquots of MSCs (2.5 × 10^5^) passage 3 were centrifuged at 1000 rpm for 5 min in 15 mL polypropylene conical tubes to form pellets, which were then cultured in high-glucose DMEM supplemented with 1% Insulin-Transferrin-Selenium, 10 nM dexamethasone, 0.17 mM ascorbic acid-2-phosphate, and 0.35 mM proline (all Sigma-Aldrich) for 14 days. Chondrogenic differentiation was histologically assessed by embedding micro-masses in optimal cutting temperature (OCT) compound and freezing, before cryosectioning at a thickness of 7 μm. Sections were stained with alcian blue (pH 2.5) at days 3, 7, and 14. For adipogenic and osteogenic differentiation, progenitor cells were plated with a density of 5 × 10^4^ cells in 6-well plates. To induce adipogenic differentiation, cells were cultured with high-glucose DMEM-medium containing 10% FCS and adipogenic supplements (1 µM dexamethasone, 200 µM indomethacin, and 50 µM 3-isobutyl-1–methylxanthine; all Sigma-Aldrich) for 14 days. Intracellular lipid vacuoles in adipogenic cultures were visualized using Oil Red O staining (Sigma-Aldrich). To induce osteogenic differentiation, cells were cultured with low-glucose DMEM-medium containing 10% FCS and osteogenic supplements (10 nM dexamethasone, 50 mM l-ascorbic acid-2-phosphate, 10 mM-glycerophosphate; all Sigma-Aldrich). Cells were cultured for 18 days, and medium was changed every other day. Osteogenic cells were observed by staining mineralized matrix components using Alizarin red.

### 3.5. Real-Time Reverse Transcription-Polymerase Chain Reaction (RT-PCR)

In accordance with the supplier’s protocol, total RNA was extracted from cultured cells using TRIzol reagent. Reverse transcription was performed by M-MLV Reverse Transcriptase (TaKaRa Biotechnology, Seoul, Kerea), according to the supplier’s specifications. Briefly, first-strand cDNA was synthesized at 37 °C for 1 h in 20 μL of reaction mixture. Quantitative real-time PCR (qRT-PCR) was carried out in a 25 μL volume container, using SYBR Green PCR Master Mix. The template source was either purified DNA standard or 5 ng cDNA. To amplify the materials, primer sequences detailed in Table 1 were used. As an internal control to standardize mRNA levels, β-actin was amplified. Relative expression of the target genes in the study samples was calculated by the comparative threshold (*C*_t_) method. The cycle of threshold (*C*_t_) for each sample was averaged and normalized to glyceraldehyde-3-phosphate dehydrogenase (GAPDH). The results were then analyzed by comparative ΔΔ*C*_t_ method (2^(−ΔΔ*C*t)^) for relative quantification of gene expression.

**Table 1:** Primer design for quantitative RT-PCR analysis.

mRNA	Primers	Annealing Tm (Cycle)
SOX9	Fw: 5'-ATCTGAAGAAGGAGAGCGAG-3'	58 °C (32)
	Rv: 5'-TCAGAAGTCTCCAGAGCTTG-3'	
Col2α1	Fw: 5'-AACACTGCAACGTCCAGAT-3'	58 °C (32)
	Rv: 5'-CTGCAGCACGGTATAGGTGA-3'	
Cartilage link protein	Fw: 5'-GCGTCCGCTACCCCATCTCTA-3'	55 °C (32)
	Rv: 5'-CTCTAAGGGCACATTCACTT-3'	
Aggrecan	Fw: 5'-GAGGTCGTGGTGAAAGGTGT-3'	58 °C (32)
	Rv: 5'-GTGTGGATGGGGTACCTGAC-3'	
ADAMS5	Fw: 5'-TGTCCTGCCAGCGGATGT-3'	58 °C (30)
	Rv: 5'-ACGGAATTACTGTACGGCCTACA-3'	
MMP-1	Fw: 5'-TCAGTTCGTCCTCACTCCAG-3'	58 °C (30)
	Rv: 5'-TTGGTCCACCTGTCATCTTC-3'	
MMP-13	Fw: 5'-GATAAAGACTATCCGAGAC-3'	58 °C (30)
	Rv: 5'-CGAACAATACGGTTACTC-3'	
TGF-β	Fw: 5'-CATCTGGAGCCTGGATACACAGT-3'	56 °C (30)
	Rv: 5'-GAAGCGCCCGGGTTGT-3'	
BMP-2	Fw: 5'-CGGGAACAGATACAGGAAGC-3'	60 °C (30)
	Rv: 5'-GCTGTTTGTGTTTGGCTTGA-3'	
BMP-4	Fw: 5'-GAGTATCTAGCTTGTCTCCCC-3'	56 °C (30)
	Rv: 5'-TCAGGTATCAAACTAGCATGG-3'	
β-actin	Fw: 5'-GCTCTCCAGAACATCACTCCTGCC-3'	58 °C (30)
	Rv: 5'-CGTTGTCATACCAGGAAATGAGCTT-3'	

Fw, forward; Rv, reverse; Tm, temperature.

### 3.6. Enzyme-Linked Immunosorbent Assay (ELISA)

The levels of TGF-β, BMP-2, and BMP-4 in conditioned media of MSCs at day 7 were measured using ELISA kits (R&D Systems, Minneapolis, MN, USA), according to the supplier’s instructions.

### 3.7. Colorimetric Analysis of Sulfated Glycosaminoglycan (sGAG) and Type II Collagen

Sulfated glycosaminoglycan (sGAG) levels in the culture medium were determined by measuring the amount of polyanionic material. Seven days after onset of culture, the culture medium was allowed to react with 1,9-dimethylmethylene blue. Twenty microliter samples were mixed with 100 μL of 5,6-dimethylbenzimidazole (DMB) reagents (48 mg/mL DMB, 40 mM glycine, 40 mM NaCl, 10 mM HCl, pH 3.0) for 30 min at room temperature, and the amount of sGAG was calculated by measuring the absorbance at 590 nm (Spectramax, Molecular Devices, Sunnyvale, CA, USA). All measurements were performed in quadruplicate. Quantification was performed using a standard curve of chondroitin 6-sulfate from shark cartilage (Sigma-Aldrich) in the range of 0–35 μg/mL.

Type II collagen levels in the medium were determined using the Sircol Collagen Assay (Biocolor Ltd., Valley Business Center, Northern Ireland). Seven days from onset of culture, the culture medium was mixed with Sirius red dye containing sulfonic acid (which reacts specifically with the basic side chain groups of type II collagens) for 30 min at room temperature using a mixer. After centrifuging for 10 min at 12,000 rpm, the unbound dye was removed, and the dye bound to type II collagen was quantified by measuring the absorbance at 540 nm and comparing that value to the standard concentration curve (0–200 μg/mL).

### 3.8. Western Blot Analysis

MSCs exposed to 1, 10, or 20 µM of mangiferin for 24 h were harvested and washed with cold PBS. Cells were lysed with lysis buffer (Invitrogen) containing protease inhibitors (10 µg/mL leupeptin, 10 µg/mL aprotinin, 10 µg/mL pepstatin A, and 1 mM of 4-(2-aminoethyl) benzenesulfonyl fluoride) and phosphatase inhibitors (1 mM NaF and 1 mM Na_3_VO_4_). Protein concentration was measured using a commercial protein assay (Bio-Rad, Hercules, CA, USA), with bovine serum albumin as the standard (Bio-Rad). Equal amounts of proteins (20–25 µg) were separated by 4%–12% SDS-polyacylamide gel (Invitrogen) electrophoresis was performed under reducing conditions, and transferred onto Hybond-C nitrocellulose membranes (Amersham Biosciences, Piscataway, NJ, USA) at 300 mA for 90 min. After blocking with 5% non-fat skim milk, the membrane was probed with primary antibodies for phospho-Smad2, phospho-Smad3, phospho-Smad1/5/8, Smad2, Smad3, Smad1/5/8 (Cell Signaling Technology, Beverly, MA, USA), SOX9, and β-actin (Sigma-Aldrich), as well as specific secondary antibodies. Protein expression was visualized by enhanced chemiluminescence (Amersham Corp., Arligton Heights, IL, USA), and signal was detected using Image Station 4000R (Kodak, New Haven, CT, USA).

### 3.9. siRNA Transfection

MSCs were transiently transfected with siRNAs targeting SOX9 (Cell Signaling Technology, Boston, MA, USA) in Lipofectamine 2000 (Gibco-BRL), according to the manufacturer’s protocols. Briefly, siRNA (200 nM) for SOX9 was suspended in 100 µL of Lipofectamine solution and mixed with an equal volume of serum-free DMEM. The mixture was added to 90% confluent chondrocytes from subchondral bone of rabbit cultured in six-well plates. Control siRNA (Dharmacon, Seoul, Korea) was used as a negative control. The specificity of SOX9 knockdown was confirmed by Western blot after siRNA transfection. After 6 h of incubation, transfected cells were washed twice with PBS and replenished with fresh medium in the presence or in the absence of IL-1β in a CO_2_ incubator for 2 h. After incubation, cells were treated with or without mangiferin (10 µM) for 72 h at 37 °C. Whole cell lysates were fractionated by 10% SDS-polyacrylamide gel electrophoresis and transferred onto nitrocellulose membranes. The nitrocellulose membranes were reacted with primary antibodies (1:1000 dilution) against phospho-Smad 2, phospho-Smad 3, phospho-Smad 1/5/8, Smad 2, Smad 3, Smad 1/5/8, aggrecan, and Col2α1.

### 3.10. Statistical Analysis

Results were expressed as mean ± standard error of mean (SEM). calculated from the specified number of experiments. Differences between groups were analyzed using one-way ANOVA. In the case of two groups, Student’s *t*-test was used. Statistical significance was assumed at a *p* value of <0.05. Data were obtained from three independent experiments.

## 4. Conclusions

**Figure 6:**
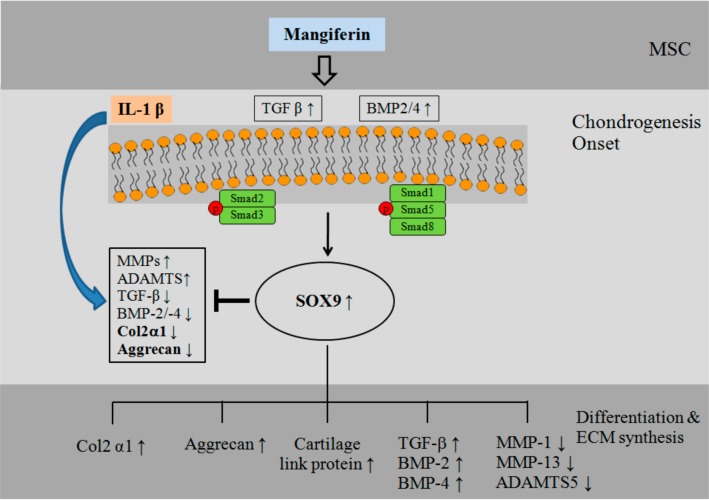
Schematic model of the pathways by which mangiferin stimulates SOX-9, a key transcription factor of TGF-β/BMPs-induced chondrogenesis, and activates the phosphorylation of Smad 1/5/8 and Smad 2/3 signaling pathways. → activation; ┤ inhibition; ↑ increase; ↓ decrease.

We observed that mangiferin induces chondrogenic differentiation in subchondral MSCs by enhancing the expression of chondrogenic markers (Col2α1, SOX9, cartilage link protein, and aggrecan) and TGF-β/BMPs. We also observed that mangiferin has chondroprotective effects, by stimulating the induction of anabolic genes (BMP-2, BMP-4, and TGF-β) and inhibiting the expression of catabolic genes (MMP-1, MMP-13, and ADAMS-5), in IL-1β-stimulated MSCs, and that these effects are mediated through the phosphorylation of the Smad 2, Smad 3, Smad 1/5/8, and SOX9 signaling pathways (Figure 6).

In conclusion, mangiferin has chondrogenic differentiation-promoting effects on MSCs from subchondral bone and regenerative effects in IL-1β-induced MSCs. We suggest that this mechanism may represent a strategy for the prevention and treatment of cartilage damage.

## References

[1] Merashly M., Uthman I. (2012). Management of knee osteoarthritis: An evidence-based review of treatment options. J. Med. Liban..

[2] Johanne M.P., Christelle B., Jean-Pierre P., Peter J.R. (2008). Cartilage in normal and osteoarthritis Conditions. Best Pract. Res. Clin. Rheumatol..

[3] Castañeda S., Roman Blas J.A., Largo R., Herrero Beaumont G. (2012). Subchondral bone as a key target for osteoarthritis treatment. Biochem. Pharmacol..

[4] Lajeunesse D. (2004). The role of bone in the treatment of osteoarthritis. Osteoarthr. Cartil..

[5] Pelletier J.P., Pelletier J.M., Raynauld J.P. (2006). Most recent development in strategies to reduce the progression of structural changes in osteoarthritis: Today and tomorrow. Arthritis Res. Ther..

[6] Neumann K., Dehne T., Endres M., Erggrlet C., Kaps C., Ringe J., Stitinger M. (2008). Chondrogenic differentiation capacity of human mesenchymal progenitor cells derived from subchondral cortico-spongious bone. J. Orthop. Res..

[7] De Vries van Melle M.L., Narcisi R., Kops N., Koevoet W.J., Bos P.K., Murphy J.M., Verhaar J.A., van der Kraan P.M., van Osch G.J. (2014). Chondrogenesis of mesenchymal stem cells in an osteochondral environment is mediated by the subchondral bone. Tissue Eng. Part A.

[8] Shapiro F., Koide S., Glimcher M.J. (1993). Cell origin and differentiation in the repair of full thickness defects of articular cartilage. J. Bone Jt. Surg. Am..

[9] Huh J.E., Cho Y.J., Yoo M.C., Baek Y.H., Lee J.D., Choi D.Y., Park D.S. (2006). Induction of effective osteogenesis by mesenchymal stem cells from the human subchondral bone. J. Korean Acupunct. Moxibustion Soc..

[10] Huh J.E., Park Y.C., Seo B.K., Lee J.D., Baek Y.H., Choi D.Y., Park D.S. (2013). Cartilage protective and chondrogenic capacity of WIN-34B, a new herbal agent, in the collagenase-induced osteoarthritis rabbit model and in progenitor cells from subchondral bone. Evid. Based Complement. Altern. Med..

[11] Yu D.A., Han J., Kim B.S. (2012). Stimulation of chodrogenic differentiatoion of mesenchymal stem cells. Int. J. Stem Cells.

[12] Fortier L.A., Barker J.U., Strauss E.J., McCarrel T.M., Cole B.J. (2011). The role of growth factors in cartilage repair. Clin. Orthop. Relat. Res..

[13] Zuscik M.J., Ma L., Buckley T., Puzas J.E., Drissi H., Schwarz E.M., O’Keefe R.J. (2007). Lead induces chondrogenesis and alters transforming growth factor-β and bone morphogenetic protein signaling in mesenchymal cell populations. Environ. Health Perspect..

[14] Kawamura I., Maeda S., Imamura K., Setoguchi T., Yokouchi M., Ishidou Y., Komiya S. (2012). SnoN suppresses maturation of chondrocytes by mediating signal crosstalk between transforming growth factor-β and bone morphogenetic protein pathways. J. Biol. Chem..

[15] Shi S., Chan A.G., Mercer S., Eckert G.J., Trippel S.B. (2014). Endogenous *versus* exogenous growth factor regulation of articular cartilage. J. Orthop. Res..

[16] Bell D.M., Leung K.K., Wheatley S.C., Ng L.J., Zhou S., Ling K.W., Sham M.H., Koopman P., Tam P.P., Cheah K.S. (1997). SOX9 directly regulates the type-II collagen gene. Nat. Genet..

[17] Pan Q., Yu Y., Chen Q., Li C., Wu H., Wan Y., Ma J., Sun F. (2008). SOX9, a key transcription factor of bone morphogenetic protein-2-induced chondrogenesis, is activated through BMP pathway and a CCAAT box in the proximal promoter. J. Cell. Physiol..

[18] García Rivera D., Delgado R., Bougarne N., Haegeman G., Berghe W.V. (2011). Gallic acid indanone and mangiferin xanthone are strong determinants of immunosuppressive anti-tumour effects of *Mangifera indica* L. bark in MDA-MB231 breast cancer cells. Cancer Lett..

[19] Das J., Ghosh J., Roy A., Sil P.C. (2012). Mangiferin exerts hepatoprotective activity against d-galactosamine induced acute toxicity and oxidative/nitrosative stress via Nrf2-NFκB pathways. Toxicol. Appl. Pharmacol..

[20] De A., Chattopadhyay S. (2009). The variation in cytoplasmic distribution of mouse peritoneal macrophage during phagocytosis modulated by mangiferin, an immunomodulator. Immunobiology.

[21] Ghosh M., Das J., Sil P.C. (2012). D(+) galactosamine induced oxidative and nitrosative stress-mediated renal damage in rats via NF-κB and inducible nitric oxide synthase (iNOS) pathways is ameliorated by a polyphenol xanthone, mangiferin. Free Radic. Res..

[22] Ang E., Liu Q., Qi M., Liu H.G., Yang X., Chen H., Zheng M.H., Xu J. (2011). Mangiferin attenuates osteoclastogenesis, bone resorption, and RANKL-induced activation of NF-κB and ERK. J. Cell. Biochem..

[23] Lefebvre V., Behringer R.R., de, Crombrugghe B. (2001). L-SOX5, SOX6 and SOX9 control essential steps of the chondrocyte differentiation pathway. Osteoarthr. Cartil..

[24] Ng L.J., Wheatley S., Muscat G.E., Conway-Campbell J., Bowles J., Wright E., Bell D.M., Tam P.P., Cheah K.S., Koopman P. (1997). SOX9 binds DNA, activates transcription, and coexpresses with type II collagen during chondrogenesis in the mouse. Dev. Biol..

[25] Akiyama H., Kim J.E., Nakashima K., Balmes G., Iwai N., Deng J.M., Zhang Z., Martin J.F., Behringer R.R., Nakamura T. (2005). Osteo-chondroprogenitor cells are derived from SOX9 expressing precursors. Proc. Natl. Acad. Sci. USA.

[26] DeLise A.M., Fischer L., Tuan R.S. (2000). Cellular interactions and signaling in cartilage development. Osteoarthr. Cartil..

[27] Kawakami Y., Rodriguez-Leon J., Belmonte J.C. (2006). The role of TGF-βs and SOX9 during limb chondrogenesis. Curr. Opin. Cell Biol..

[28] Derfoul A., Perkins G.L., Hall D.J., Tuan R.S. (2006). Glucocorticoids promote chondrogenic differentiation of adult human mesenchymal stem cells by enhancing expression of cartilage extracellular matrix genes. Stem Cells.

[29] Mehlhorn A.T., Niemeyer P., Kaschte K., Muller L., Finkenzeller G., Hartl D., Sudkamp N.P., Schmal H. (2007). Differential effects of BMP-2 and TGF-β1 on chondrogenic differentiation of adipose derived stem cells. Cell Prolif..

[30] Tetlow L.C., Adlam D.J., Woolley D.E. (2001). Matrix metalloproteinase and proinflammatory cytokine production by chondrocytes of human osteoarthritic cartilage: Associations with degenerative changes. Arthritis Rheumatol..

[31] Fernandes J.C., Martel-Pelletier J., Pelletier J.P. (2002). The role of cytokines in osteoarthritis pathophysiology. Biorheology.

[32] Joos H., Wildner A., Hogrefe C., Reichel H., Brenner R.E. (2013). Interleukin-1β and tumor necrosis factor alpha inhibit migration activity of chondrogenic progenitor cells from non-fibrillated osteoarthritic cartilage. Arthritis Res. Ther..

[33] Blaney Davidson E.N., van der Kraan P.M., van den Berg W.B. (2007). TGF-β and osteoarthritis. Osteoarthr. Cartil..

[34] Diao H., Wang J., Shen C., Xia S., Gou T., Donq L., Zhang C., Chen J., Zhang J. (2009). Improved cartilage regeneration utilizing mesenchymal stem cells in TGF-β1 gene-activated scaffolds. Tissue Eng. Part A.

[35] Gouttenoire J., Valcourt U., Ronziere M.C., Aubert Foucher E., Mallein Gerin F., Herbage D. (2004). Modulation of collagen synthesis in normal and osteoarthritic cartilage. Biorheology.

[36] Chung C., Burdick J.A. (2008). Engineering cartilage tissue. Adv. Drug Deliv. Rev..

[37] Glasson S.S. (2007). *In vivo* osteoarthritis target validation utilizing genetically-modified mice. Curr. Drug Targets.

[38] Derynck R., Zhang Y.E. (2003). Smad-dependent and Smad-independent pathways in TGF-β family signalling. Nature.

[39] Schmierer B., Hill C.S. (2007). TGF-β-Smad signal transduction: Molecular specificity and functional flexibility. Nat. Rev. Mol. Cell Biol..

[40] Miyazono K., Kamiya Y., Morikawa M. (2010). Bone morphogenetic protein receptors and signal transduction. J. Biochem..

[41] Xiao Y.T., Xiang L.X., Shao J.Z. (2007). Bone morphogenetic protein. Biochem. Biophys. Res. Commun..

[42] Furumatsu T., Tsuda M., Taniguchi N., Tajima Y., Asahara H. (2005). Smad3 induces chondrogenesis through the activation of SOX9 via CREB-binding protein/p300 recruitment. J. Biol. Chem..

[43] Sekiya I., Tsuji K., Koopman P., Watanabe H., Yamada Y., Shinomiya K., Nifuji A., Noda M. (2000). SOX9 enhances aggrecan gene promoter/enhancer activity and is up-regulated by retinoic acid in a cartilage-derived cell line, TC6. J. Biol. Chem..

